# Atomic scale determination of magnetism and stoichiometry at the La_0.7_Sr_0.3_MnO_3_/SrTiO_3_ interface: investigation of inverse hysteresis

**DOI:** 10.1038/s41427-025-00590-y

**Published:** 2025-03-07

**Authors:** Gyanendra Panchal, Federico Stramaglia, Pawan Kumar, Enrico Schierle, Klaus Habicht, Carlos A. F. Vaz, Katharina Fritsch

**Affiliations:** 1 Swiss Light Source, Paul Scherrer Institut, Villigen, Switzerland; 2 Department Dynamics and Transport in Quantum Materials, Helmholtz-Zentrum Berlin für Materialien und Energie, Berlin, Germany; 3 Department of Materials Science and Engineering, University of Pennsylvania, Philadelphia, PA USA; 4 Inter-university Microelectronics Center (IMEC), Leuven, Belgium; 5 Institute for Quantum Phenomena in Novel Materials, Helmholtz-Zentrum Berlin für Materialien und Energie, Berlin, Germany; 6 Institut für Physik und Astronomie, Universität Potsdam, Potsdam, Germany

**Keywords:** Surfaces, interfaces and thin films, Magnetic properties and materials

## Abstract

Controlling the correlations and electronic reconstruction at the interface of transition metal oxide heterostructures provides a new pathway for tuning their unique physical properties. Here, we investigate the effects of interfacial nonstoichiometry and vertical phase separation on the magnetic properties and proximity-induced magnetism of epitaxial La_0.7_Sr_0.3_MnO_3_ (LSMO)/SrTiO_3_(001) oxide heterostructures. We also reinvestigate the recently observed inverse hysteresis behavior reported for this system, which we find emanates from the remanent field of the superconducting solenoid and not from antiferromagnetic intra-layer exchange coupling in low coercivity LSMO thin films. Combined atomically resolved electron energy loss spectroscopy, element-specific X-ray magnetic circular dichroism, and interface-sensitive polarized soft X-ray resonant magnetic reflectivity show the formation of a Mn^3+^-enriched interfacial LSMO layer, of a Ti^3+^-derived magnetic interface layer coupled ferromagnetically to La_0.7_Sr_0.3_MnO_3_, together with a small density of O-vacancies at the interface. These results not only advance the understanding of the magnetism and spin structure of correlated oxide interfaces but also hold promise for practical applications, especially in devices where the performance relies on the control and influence of spin polarization currents by the interfacial spin structure.

## Abstract (web summary)

Controlling stoichiometry and electronic reconstruction at the interface of transition metal oxide heterostructures offers a new way to tune their unique properties, leading to behaviors distinct from the bulk components. Using atomically resolved scanning transmission electron microscopy, element specific x-ray resonant magnetic reflectivity and x-ray magnetic circular dichroism, we have demonstrated that the presence of local oxygen deficiency across the interface and charge transfer to the empty conduction band of SrTiO_3_ at the interface are the primary drivers for the modified interfacial magnetism in the manganite thin films and Ti^3+^ induced ferromagnetism at the La_0.7_Sr_0.3_MnO_3_/SrTiO_3_ interface.

## Graphical abstract

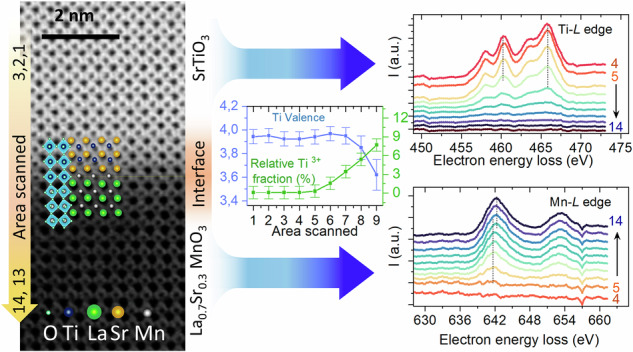

## Introduction

Symmetry breaking at the interface of transition metal oxide (TMO) thin films is known to lead to unexpected electronic and magnetic properties by triggering electronic and structural reconstructions^1^. Discontinuities in the electronic state, atomic structure, chemical valence and orbital occupancy at the interface play important roles in modifying not only the lattice structure and charge transfer at the interface but also in modifying exchange interactions. For example, at the heterointerface, the charge transfer caused by a difference in chemical potential or by the screening of local dipoles can modify the valence state of TMOs^1–4^. These interfacial reconstructions and strong coupling between charge and spin in correlated electron systems yields a variety of new physical properties and functionalities, such as high-temperature superconductivity, ferroelectricity, magnetoelectric coupling and exotic complex magnetic ordering, which are absent in their bulk form^5–8^. Additionally, nonstoichiometry, which commonly arises in materials at the interface of heterostructures, yield surprising electronic and magnetic properties via unintentional doping, defects, or electronic reconstruction induced via polar mismatch close to the TMO interface^9^. Examples include the formation of a highly mobile 2D electron gas^10,11^ or the observation of in-plane ferromagnetic order^12^ at the interface between two insulating LaAlO_3_–SrTiO_3_ oxides.

The mixed-valence manganite La_1−*x*_Sr_*x*_MnO_3_ is an important TMO system that has been extensively investigated in thin film form for its room-temperature half-metallicity^13,14^ and large magnetoresistance^15^ as a system for desirable applications in spintronic devices.

However, La_0.67_Sr_0.33_MnO_3_/SrTiO_3_ heterostructures display reduced magnetization^16^ and orientationally modified interfacial magnetism^17^. Notably, the magnetic properties of manganite thin films are highly sensitive to double exchange interactions, superexchange interactions and Jahn–Teller distortions, which can be altered via strain^18,19^, metal-oxygen bond length/angle^20,21^, and modified formal charge states of the interfacial ions^22,23^.

Several studies have focused on understanding the interface magnetism in manganite thin films grown on SrTiO_3_ (STO) substrates using different techniques, such as polarized neutron reflectometry (PNR) on La_0.8_Sr_0.2_MnO_3_/STO^22^ and surface sensitive X-ray magnetic circular dichroism (XMCD) on La_0.7_Sr_0.3_MnO_3_(LSMO)/STO heterostructures^24^, to show that a variation in the Mn^3+^/Mn^4+^ ratio is important for the formation of an intermediate LSMO layer close to the interface.

In the last decade, several studies have addressed exotic magnetization reversal, including negative magnetic remanence and inverse hysteresis [positive (negative) coercivity in the descending (ascending) field cycle] mediated by intralayer exchange coupling within the LSMO layer of LSMO/STO(001) heterostructures. Recently, Mottaghi et al.^25^ reported a negative magnetic remanent state (inverse hysteresis) in a 7.6 nm thick LSMO film in a temperature range from 240 to 290 K. Saghayezhian et al.^26^ reported spontaneous magnetic reversal and inverse hysteresis in La_2*/*3_Sr_1*/*3_MnO_3_/STO heterostructures via DC magnetization and, on the basis of the results of atomically resolved transmission electron microscopy/spectroscopy, suggested that a structural gradient in the oxygen octahedral network and oxidation state variation were responsible for the inverse hysteresis. These authors also showed that a specific oxygen partial pressure (40 mTorr) and a critical thickness [15 unit cell (u.c.)] are required for such complex magnetic behavior^27^. Recently, Guo et al.^28^ reported inverse hysteresis in an LSMO/PZT heterostructure as well.

Interestingly, in all the above mentioned studies^26–28^, the magnetic properties were measured by superconducting quantum interference device (SQUID) magnetometry, and the coercivities of the measured samples were approximately 20 Oe or less, which is within the remanence limit of the superconducting solenoid. In such measurement systems, the magnetic field is not directly measured, and the presence of a remanent field can cause inaccuracies or errors up to 20–40 Oe and measurement artifacts, including inverse hysteresis^29^. Therefore, the claim of inverse hysteresis induced via antiferromagnetic (AFM) intra-layer exchange coupling in thin films with low coercivity ( < 30 Oe) remains controversial. Direct evidence for magnetic intra-layer exchange coupling, such as elemental magnetic depth profiling, has not been obtained in the above studies. Thus, the origin of such unusual magnetization reversal remains unclear.

In this work, we investigate the hysteresis behavior of different epitaxial La_0.7_Sr_0.3_MnO_3_/STO(001) heterostructures by detailed DC magnetization measurements, carefully considering the remanence effect of the superconducting solenoid. We discuss the microscopic origin of the electronic and magnetic properties at the interface and inter-layer close to the STO substrate in terms of the combined effects of structural reconstruction and modified Mn valence and the presence of Ti^3+^-induced magnetism at the LSMO/STO interface. From element-specific soft X-ray resonant magnetic reflectivity (XRMR) at the Mn and Ti L-edges, X-ray magnetic circular dichroism and high-resolution electron energy loss spectroscopy (EELS), we demonstrate that the combined effect of the Ti^3+^ states and the modified ratio of Mn^3+^/Mn^4+^ close to the interface plays a vital role in modifying the interface magnetism and results in the suppression of the magnetic dead layer and proximity-induced ferromagnetism in the Ti ions.

## Results and Discussion

### Structural properties

**Fig. 1:**
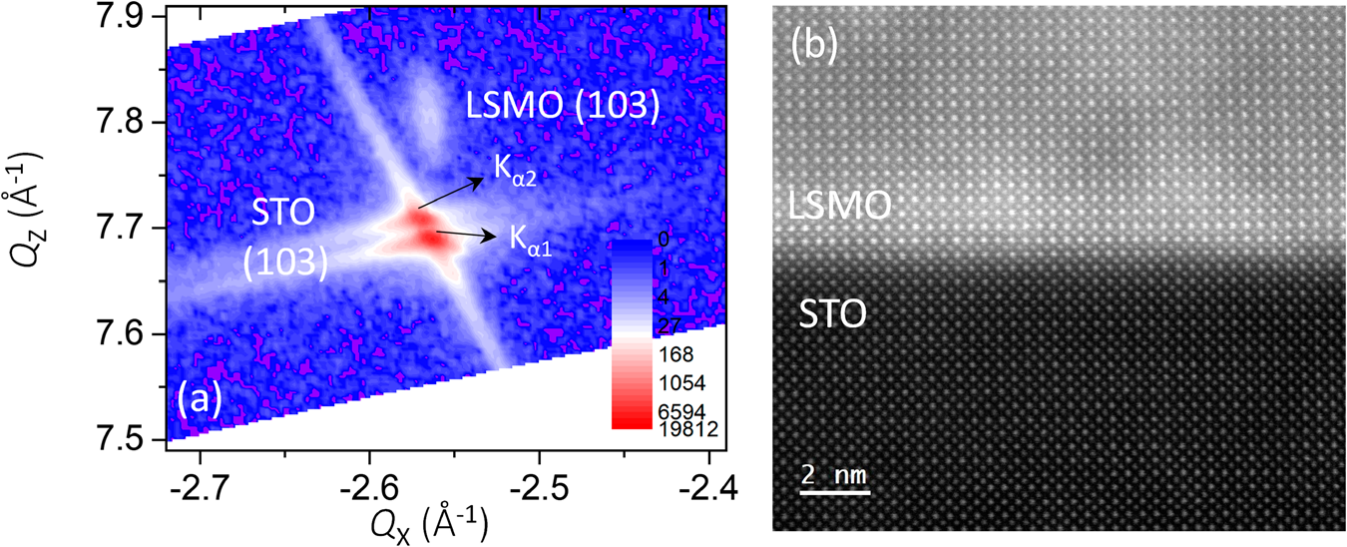
Structural characterization of the LSMO/STO interface. **a** Reciprocal space mapping around the asymmetric (103) Bragg reflection of 20 nm La_0.7_Sr_0.3_MnO_3_ grown on a SrTiO_3_(001) substrate. The arrows indicate the splitting of the substrate Bragg peak due to Cu-K_*α*1_ and Cu-K_*α*2_ radiation from the X-ray source. **b** An atomically resolved aberration-corrected STEM image of the cross-sectional view of the La_0.7_Sr_0.3_MnO_3_/SrTiO_3_ interface.

Single-phase LSMO thin films (pseudocubic bulk lattice parameter = 3.869 Å)^19,30^ were grown on STO(001) substrates by pulsed laser deposition (PLD). Reciprocal space mapping (RSM) around the asymmetric (103) plane, presented in Fig. 1a, shows that the LSMO thin film is epitaxial and fully strained to the STO substrate (same *Q*_*x*_ for both the film and the substrate). The average calculated in-plane and out-of-plane lattice parameters are *a* = *b* = 3.902 Å and *c* = 3.843 Å, respectively (*c*/*a* = 0.984, corresponding to in-plane tensile strain). The aberration-corrected high angle annular dark field (HAADF)-scanning transmission electron microscopy (STEM) image in Fig. 1b shows an atomically sharp LSMO/STO interface and confirms that the LSMO film is fully strained to the substrate with minimal defect density.

### Magnetic properties

**Fig. 2:**
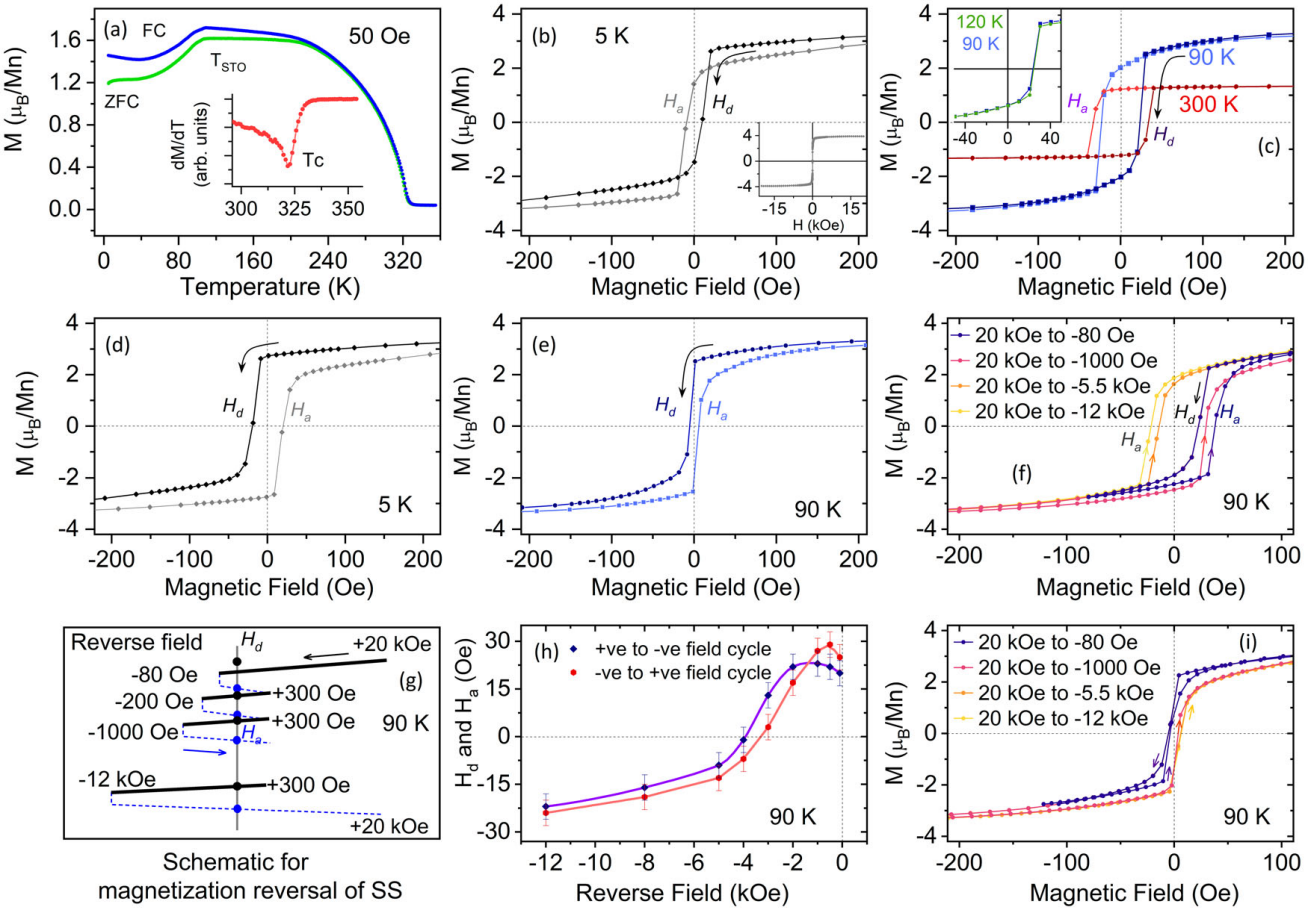
Bulk magnetometry characterization. **a** FC and ZFC temperature-dependent in-plane magnetization *M-T* of the LSMO/STO heterostructure with a 50 Oe cooling field; the inset shows d*M*/d*T*. Magnetization hysteresis before remanent field correction at (**b**) 5 K, (**c**) 90, and 300 K [inset in (**c**) shows the descending field cycle at 90 and 120 K]. Magnetization hysteresis after correcting the remanent field of the superconducting solenoid using a Pd reference sample at (**d**) 5 K and (**e**) 90 K. **f** Minor hysteresis loop (before remanent field correction) measured using different field cycles at 90 K. Schematic of the magnetic field cycling sequence (**g**) and calculated remanent field (**h**) of the superconducting solenoid at 90 K using the Pd reference sample. **i** Minor hysteresis loop after remanent field correction at 90 K.

Temperature dependent field-cooled (FC) and zero field-cooled (ZFC) in-plane magnetizations under a 50 Oe magnetic field measured by SQUID are shown in Fig. 2a. From the magnetization versus temperature (*M–T*) behavior, we estimate that the paramagnetic to ferromagnetic ordering temperature (*T*_C_) of the thin film is 323 K [see the inset of Fig. 2a], which is consistent with the previously reported^31^ *T*_C_ of strained LSMO. The anomaly in the *M–T* curve of LSMO around 105 K (*T*_sto_) is attributed to the cubic to antiferrodistortive phase transition of the underlying STO substrate^6,32–36^. When the change in the lattice parameter is on the order of 0.1%, the epitaxially grown magnetoelastically coupled LSMO thin film is extremely sensitive to such breaking of symmetry, as shown by the low-field *M–T* data in Fig. 2a, which is suppressed at a high measuring field. Figure 2b, c show the magnetic hysteresis loops at different temperatures measured under zero-field cooled conditions. The saturation magnetic moment of the LSMO layer is 3.85 *µ*_B_/Mn [see the inset of Fig. 2b], similar to the bulk value, indicating that the film is fully magnetically polarized with no interfacial dead layer. We discuss this aspect later in the EELS section.

Furthermore, it is interesting to note that the magnetization hysteresis recorded at 5 K displays an inverse hysteresis behavior, namely, positive *H*_d_ and negative coercivity *H*_a_ in descending (positive to negative) and ascending (negative to positive) field cycles, respectively, with very small coercivity (∼12 Oe), as shown in Fig. 2b. Such negative remanence behavior has been previously reported for multilayer systems, typically resulting from interlayer AFM exchange coupling between magnetic layers possessing different uniaxial anisotropies, for example, [Co-Pt]/[Gd-Pt]^37^ (coercivity *>* 1 kOe) and Co/Mn/Co^38^ (coercivity *>* 100 Oe), and also recently in LSMO/STO heterostructures^25,26^, as discussed in the introduction.

This inverse hysteresis behavior in the magnetization reversal is also observed across the structural phase transition temperature (105 K) of STO, as shown in Fig. 2c, with no significant changes in the magnetization response, which is in agreement with previous observations^36^ [inset Fig. 2c], indicating that the inversion is not related to the structural phase transition of the STO substrate^31,39^. We find that the negative coercivity of the heterostructure increases with increasing temperature and persists up to room temperature, a behavior opposite to that expected from the intrinsic magnetic behavior of ferromagnetic materials^40,41^, where the thermal excitation tends to favor the formation of reverse domains and domain wall motion. The behavior of the inverse hysteresis as a function of the angle *ϕ* between the sample plane and the applied magnetic field is shown in Fig. S1 of the supplementary information. Upon rotation of the sample in the out-of-plane direction, the coercivity of the sample increases (up to 50 Oe), and the *M‒H* loop transforms to a normal hysteresis loop (Fig. S1 in the supplementary section).

We note that the in-plane coercivity of the LSMO layer measured here is approximately 20 Oe or less, as noted in recent publications^26–28^, which is below the remanence limit (20*‒*40 Oe) of the superconducting solenoid of SQUID magnetometers. This can potentially lead to measurement artifacts, including inverse hysteresis^29^. Therefore, to exclude the effect of the remanent field of the superconducting solenoid, we performed detailed magnetization measurements on a paramagnetic palladium (Pd) reference sample to calculate the remanent field of the superconducting solenoid. Interestingly, after the remanent field is corrected, the inverse hysteresis recorded at 5 and 90 K transforms into normal *M‒H* curves, with a wasp-waisted or pinched shape, as shown in Fig. 2d, e^31,42,43^. We confirm this result by measuring minor loops at 90 K after applying +20 kOe for a set of increasing reverse fields from –80 Oe to –12 kOe. The results are presented in Fig. 2f. We find that, as we increase the reverse field, the coercivity for the ascending branch *H*_a_ decreases and flips sign at a certain reverse field (between –1 and –5.5 kOe), similar to the positive exchange bias reported in the LSMO/PZT heterostructure^28^. To confirm whether this behavior is intrinsic to the system, we used the measurement protocol schematically shown in Fig. 2g on a Pd reference sample to determine the variation in the remanent field of the superconducting solenoid with increasing reverse field strength. We find that the remanent field remains the same in both descending ( + ve to –ve) and ascending (–ve to +ve) loop branches to within ± 4 Oe. Furthermore, we see that the remanent field remains approximately constant up to -2 kOe and flips sign for reverse fields exceeding –4 kOe, as shown in Fig. 2h Once the remanent field in the minor loop is corrected for, the exotic positive exchange bias behavior disappears [Fig. 2d, e, i]. These results were confirmed by performing similar measurements on a second 20 nm LSMO/STO(001) sample grown under identical conditions, where we observe a similar inverse hysteresis behavior, as well as a transition from inverse hysteresis to normal hysteresis at 30 K during cooling (260 to 5 K) [shown in Fig. S2 in the supplementary section], which transforms into a normal hysteresis behavior after correcting the remanent field of the superconducting solenoid, and displays an increase in coercivity with decreasing temperature, characteristic of ferromagnets (Fig. S2)^40,41^. The observation of a regular *M‒H* loop for a magnetic field applied out of plane, where the coercivity is higher than the remanent field of the solenoid, is consistent with the explanation given above. Thus, we find no evidence for the presence of inverse hysteresis in LSMO/STO and attribute its observation in low coercivity systems to artifacts from the remanent field value of the superconducting solenoid, which needs to be considered when the data are evaluated^29^.

### Electronic properties

#### Electron energy loss spectroscopy (EELS) results

**Fig. 3:**
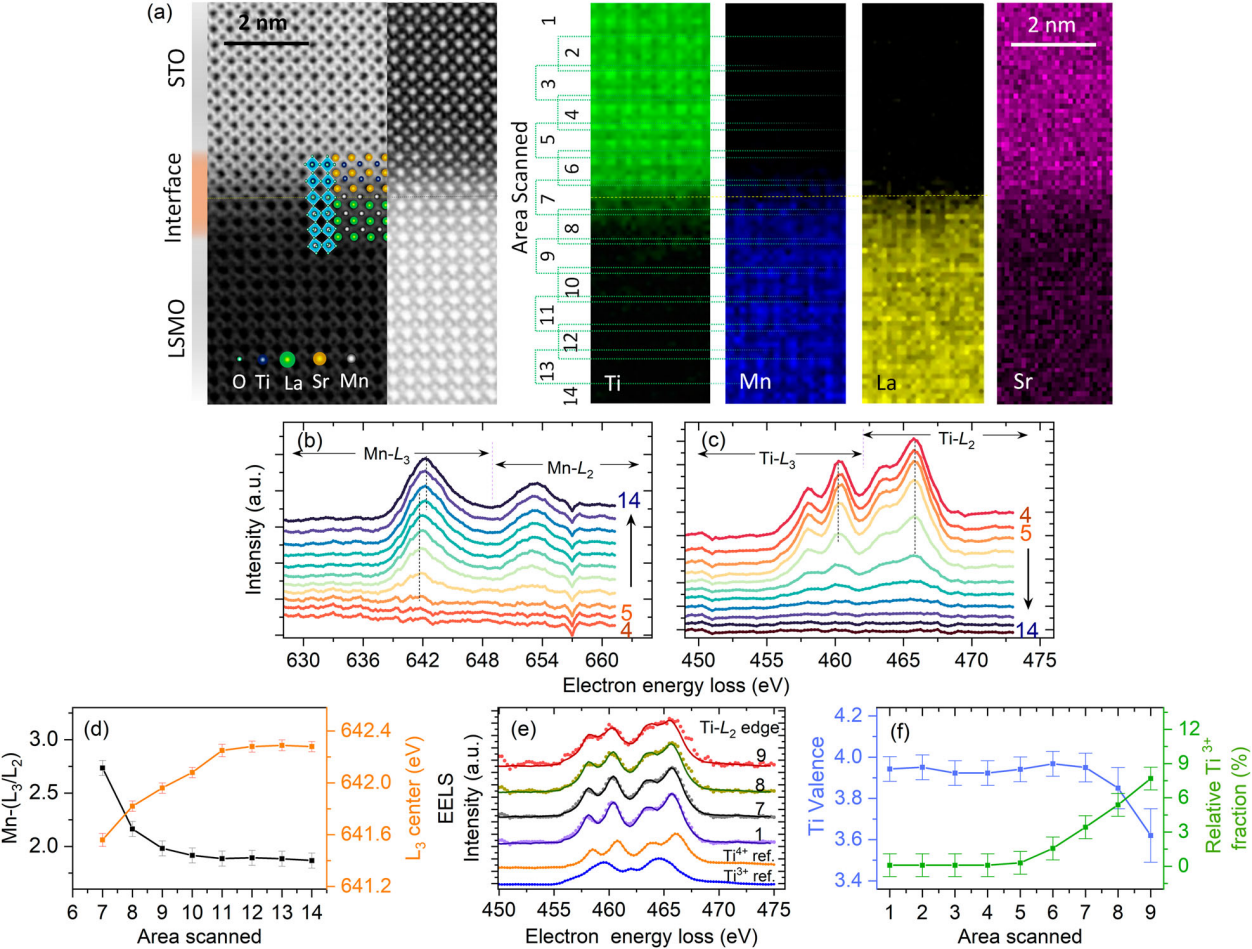
Atomically resolved spectroscopy at the LSMO/STO interface. **a** Local oxygen octahedra across the LSMO/STO heterointerface in an atomically resolved HAADF-ABF STEM image along with EELS mapping across the LSMO/STO heterointerface; the rectangular areas (numbered from 1–14) correspond to the scanned regions used to collect the EELS spectra. Layer-resolved EELS spectra at the (**b**) Mn L-edge and (**c**) Ti L-edge. (**d**) Calculated *L*_3_/*L*_2_ ratio and Mn *L*_3_ peak position as a function of the scanned area. **e** Deconvolution of Ti^3+^ and Ti^4+^ spectra from the experimental spectra via a linear sum of Ti L-edge reference spectra for Ti^4+^ (orange) and Ti^3+^ (blue) taken from Wang et al.^45^. **f** Calculated interfacial Ti valence and the relative Ti^3+^ fraction as a function of the interfacial scanned area.

To evaluate the effects of interface chemistry and charge redistribution on the electronic structure and magnetic properties across the LSMO/STO heterointerface at the atomic scale, we performed atomically resolved aberration-corrected STEM-EELS spectrum imaging at room temperature. A HAADF-STEM image and elemental EELS map of the sample are shown in Fig. 3a. The STO substrate is SrO-terminated in this particular local region. We observe the periodic octahedral tilt of LSMO close to the interface and cannot identify any strong modifications in the structure close to the interface, as shown in the HAADF image.

As we go across the interface from LSMO to STO (bottom to top), the EELS line profile gradually changes from a Mn-edge dominated spectrum to a Ti *L*_2,3_-edge dominated spectrum [Fig. 3b, c]. The variation in the spectral weight ratio of the Mn *L*_3_ and Mn *L*_2_ features clearly indicates that the stoichiometry of the LSMO thin film close to the interface along the surface normal is nonuniform (Fig. 3b). To quantitatively obtain the formal valence of Mn near the LSMO/STO interface, the ratio *L*_3_/*L*_2_ is calculated from the integrated area of individual Mn *L*_3_ and Mn *L*_2_ peaks after background subtraction following the work of Varela et al.^44^ and Wang et al.^45^. It can be seen that the *L*_3_/*L*_2_ ratio increases near the LSMO/STO interface, indicating a decrease in the Mn valence state (from a mixed Mn^3+^/Mn^4+^ state to a Mn^3+^ rich state), as shown in Fig. 3d. This observation is also consistent with the peak position shift (toward lower energy) shown by the vertical line in Fig. 3b, which we attribute either to oxygen deficiency^46,47^ or to a compensating interface charge^48^. This Mn^3+^ enriched interface prevents the formation of a dead layer^49,50^.

**Fig. 4:**
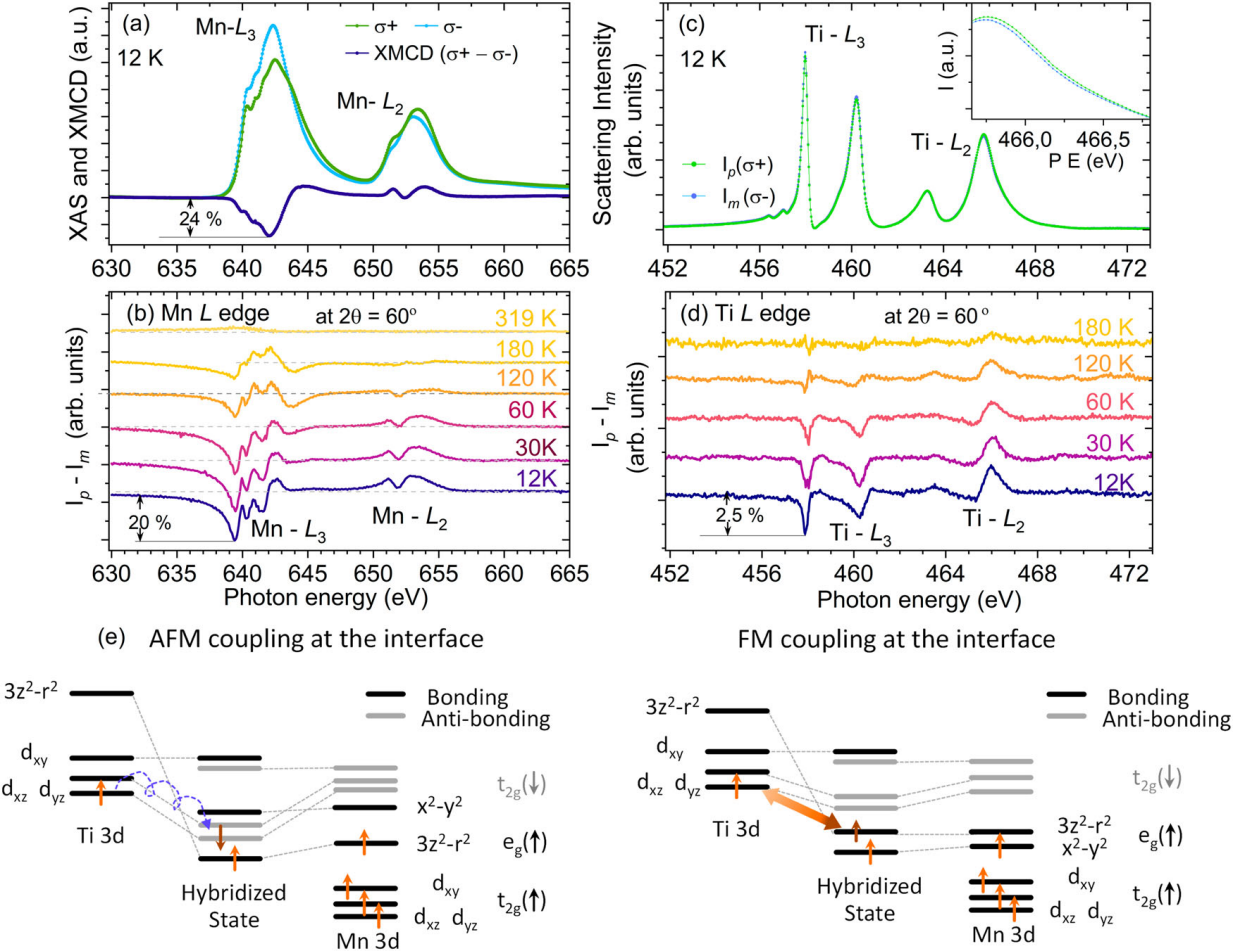
Resonant x-ray reflectivity results. **a** XAS spectra for *σ*^+^ and *σ*^−^ helicity and corresponding XMCD in TEY mode at the Mn L-edge at 12 K. **b** Temperature-dependent dichroic X-ray reflectivity (I_*p*_-I_*m*_) energy scans across the Mn L-edge. **c** X-ray reflectivity energy scan for *σ*^+^ and *σ*^−^ helicity across the Ti L-edge at 12 K. **d** Temperature-dependent dichroic X-ray reflectivity energy scans across the Ti L-edge. **e** Schematic energy level diagrams of the orbital hybridization proposed for FM and AFM spin‒spin coupling between Ti and Mn with preferentially occupied Mn 3d–*e*_*g*_ orbitals and relative occupancies of the Ti 3d-*xz*,*yz* orbitals at the electron‒doped titanate and a manganite interface in LSMO/STO heterostructure^58,63^.

We also observe a significant change in the Ti L-edge EELS spectra across the interface, namely, a reduced oxidation state of Ti along with a small Ti^3+^ fraction [Fig. 3(c)] close to the interface. The presence of Ti^3+^ can be quantitatively determined by deconvoluting the reference spectra of Ti^4+^ and Ti^3+^, as shown in Fig. 3(e), following the work of Ohtomo et al.^51^ and Wang et al.^45^ These results show that the relative fraction of Ti^3+^ gradually increases from the STO to the LSMO/STO interface, whereas the Ti^4+^ valence decreases, as shown in Fig. 3(f). The presence of Ti^3+^ (3d^1^ state) explains the origin of the unusual magnetic dichroism observed at the Ti L-edge in soft X-ray resonance reflectivity measurements (shown in Fig. 4).

In the O K-edge EELS spectra, we observe a significant modification and a small damping in the O K-edge features/fine structure (see Fig. S3 of the supplementary information) only across the interface, indicating the presence of oxygen vacancies at the interface^46^.

From these results, we can also rule out mutual charge transfer from Mn to Ti because both valence states of the Mn and Ti ions are reduced toward the interface, which we instead associate with a small density of O vacancies. Notably, the presence of oxygen vacancies at the LSMO/STO interface and reduced Mn species has been reported in other studies, such as that by Nord et al.^52^. It has also been suggested that the formation of oxygen vacancies at the interface diminishes the polar field at the interface and suppresses the mutual charge transfer from Mn to Ti^23,53^. The presence of oxygen vacancies is expected to result in electron/hole doping and affects the superexchange and double-exchange interactions at the interface. On the basis of the gradient in the Mn/Ti valence state variation and O K edge EELS, we conclude that the oxygen vacancies are confined to the interface (up to 5–6 uc) and are not induced by the O_2_ partial pressure during growth. These results imply that the charge imbalance induced by the presence of oxygen vacancies at the interface has an important role in stabilizing the modified electronic and magnetic states at the interface^48^.

#### XMCD and X-ray resonant magnetic reflectivity (XRMR) results

To directly explore the interfacial magnetism of the LSMO/STO heterostructures and the role of phase separation and off-stoichiometry on the overall magnetic and electronic properties, we carried out XRMR measurements at the Mn and Ti L-edges. XRMR is sensitive to the magnetic depth profile and provides element-specific information about the magnetic spin configuration at the LSMO/STO interface. XMCD measurements were performed in surface sensitive total electron yield (TEY) mode along with bulk sensitive scattering mode in a reflectivity geometry. The absorption spectra in TEY mode at the Mn L-edge recorded at 12 K in the field-cooled remanent state (following a similar protocol used for the SQUID measurements) exhibit a very strong characteristic XMCD signal (24%), as shown in Fig. 4a. The XMCD sum rules^54–58^ give an effective magnetic moment of *m*_eff_ = 2.22 ± 20% *µ*_*B*_/Mn (see Fig. S4 of the supplementary information), which is slightly reduced with respect to the remanent magnetic moment value, as observed by SQUID VSM measurements (Fig. 2d). This reduced magnetic moment might be attributed to reduced magnetization at the surface of the film at low fields, which strongly influences the TEY XMCD signal, as it decays exponentially with depth. To probe deeper into the LSMO layer, we measured the reflected intensity for the two helicities as a function of energy (energy scan) across the Mn L-edge at an angle of 30° from the surface, where the probing depth is limited by the X-ray attenuation length of approximately 80 nm (much larger than the film thickness). The corresponding dichroic spectra for temperatures ranging from 12 to 319 K are shown in Fig. 4b. In addition to the expected reduction in the signal amplitude with increasing temperature, we observed a strong change in the dichroic reflectivity, indicating a magnetization redistribution as a function of depth with temperature.

Furthermore, the dichroic reflectivity across the Ti L-edge energy range presented in Fig. 4c show a notable (2.5%) dichroic signal (XMCD) at 12 K, as shown in Fig. 4d, revealing the presence of magnetism^59,60^ at the STO interface and the presence of Ti^3+^ at the LSMO/STO interface, which is consistent with the observations in the EELS measurements. Owing to the limited escape depth of electrons, no TEY signal at the Ti edge could be detected. The magnetic polarization of Ti is also confirmed by the measurement of a circular dichroic signal in X-ray resonant magnetic reflectivity measurements at the Ti L edge, as shown in Fig. S5. The dichroism at the Ti L-edge is nearly eight times weaker than that at the Mn L-edge. The relative signs of the Mn L_3_-edge dichroism in the surface-sensitive TEY signal and bulk-sensitive reflectivity at the Mn and Ti L_3_-edge are the same, demonstrating that the Ti and Mn magnetic moments are oriented in the same direction. In addition, we find that the intensity of the Ti magnetic dichroic reflectivity decreases with increasing temperature and has a similar temperature dependence as the Mn dichroic reflectivity signal, demonstrating that the Ti magnetic polarization follows that of Mn, i.e., that the Ti spins are ferromagnetically coupled to the Mn spins. Overall, these results indicate that Ti magnetism has an interfacial origin and is ferromagnetically coupled to the moment of the Mn site in the nonstoichiometric Mn^3+^-rich interfacial LSMO layer close to the STO substrate^58,61^.

Garcia Barriocanal et al.^58,62^ experimentally demonstrated that, for electron-doped titanate/manganite superlattice, the sign of spin‒spin coupling interaction between the Mn and Ti spin moments at the electron-doped titanate and the manganite interface can be tuned from antiferromagnetic to ferromagnetic by the strain state of the superlattice, which is explained in terms of changes in the preferential occupation (orbital polarization) of the Mn 3d e_*g*_ orbitals and is controlled by the manganite to titanate thickness ratio *t*_r_ (*t*_LMO_*/t*_STO_). For the large *t*_r_ and relaxed LaMnO_3_ layer, these authors observed antiferromagnetic Ti–Mn magnetic coupling (schematically shown in Fig. 4e), whereas for the strained film (low *t*_r_ with 3d_*x*_^2^_−*y*_^2^ preferential occupation), they observed ferromagnetic Ti–Mn magnetic coupling at the interface^63^. In our case, the thin films are fully tensile-strained, and the interface has a similar structure. The manganese Mn 3d_*x*_^2^_−*y*_^2^ orbitals are preferentially occupied, and the hybridization between the occupied Mn 3d_*x*_^2^_−*y*_ orbitals and Ti orbitals across the interface is weak. A finite overlap between the Ti *t*_2*g*_ orbitals and the Mn d_3*z*_^2^_−*r*_^2^ orbitals resulting from the GdFeO_3_-type distortion of manganite may yield finite values of the transfer integral between the 3*z*^2^ − *r*^2^ orbitals and the active *t*_2*g*_-d_*xz*_ and d_*yz*_ orbitals. A ferromagnetic coupling between the moment at the Ti and Mn sites results from the superexchange interaction through the virtual excitation of electrons from Ti *t*_2*g*_ (d_*xz*_, d_*yz*_) to Mn d_3*z*_^2^>_−*r*_^2^, as schematically shown in Fig. 4e^58,63^.

EELS, element-specific XMCD, and magnetic reflectivity experiments show that the LSMO/STO heterostructure behaves like a trilayer system: a bulk LSMO layer, an interfacial LSMO layer and an interfacial STO layer, which are chemically and magnetically distinct from each other. We point out that, in the remanent state, the interfacial Ti magnetic moment (probed by highly sensitive XRMR) and the Mn magnetic moment (TEY absorption) in the top bulk layer are parallel to each other in contrast to the antiparallel alignment in the LSMO/STO superlattices and thin films^24,48^. However, we cannot establish from the XAS measurements the magnetic moment orientation of the Mn ions of the LSMO interlayer close to the STO and the bulk-like LSMO top layer.

**Fig. 5:**
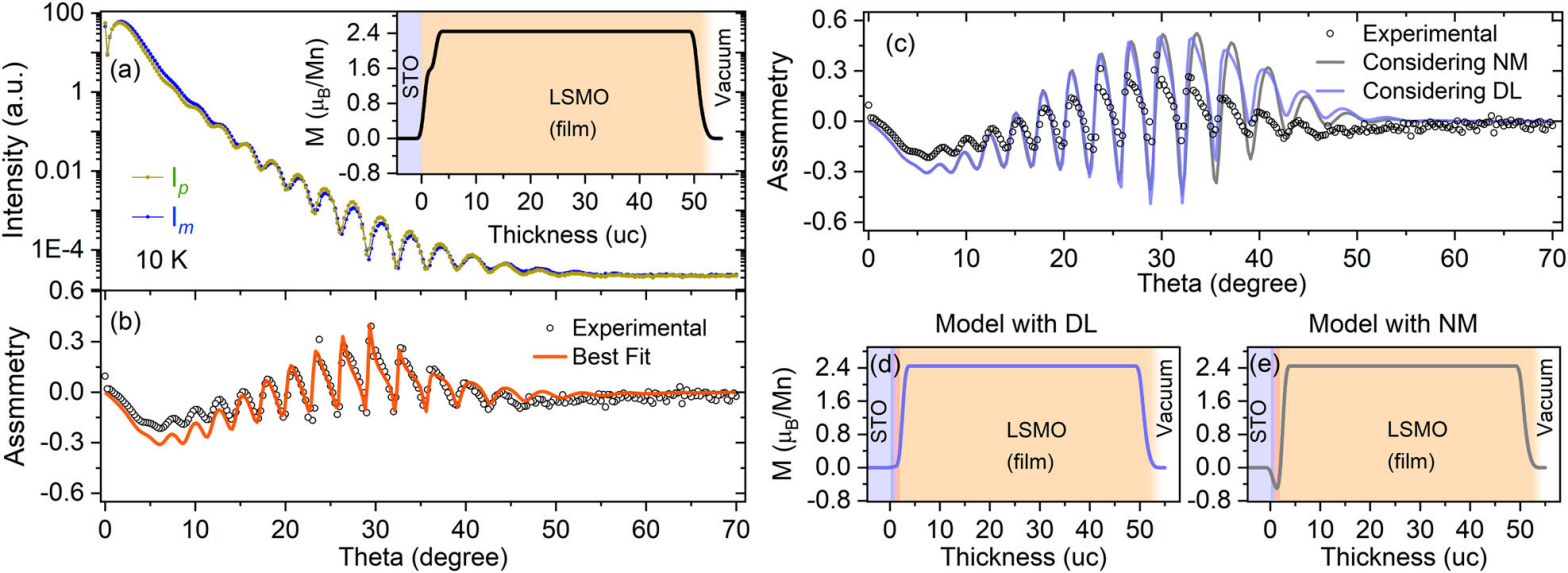
Depth profile of the magnetic interface structure. **a** Specular soft X-ray reflectivity curves I_*p*_ and I_*m*_ at the Mn L_3_-edge resonant energy of 642 eV. **b** Experimental and calculated magnetic asymmetry at 10 K. The inset of (**a**) represents the element-specific magnetization depth profile for the Mn ion derived from the best fit of the magnetic asymmetry. **c** Experimental and calculated magnetic asymmetry with models considering the dead layer (DL) and negative magnetization (NM) at the LSMO/STO interface (up to 3 uc) and their respective magnetic depth profiles (**d**, **e**) for the Mn ion, derived from the refinement of the magnetic asymmetry.

To obtain this information, we carried out polarized soft X-ray resonant magnetic reflectivity measurements to probe the magnetization profile^64–69^ across the entire sample thickness for the Mn ions. X-ray resonant magnetic reflectivity curves *I*_p_ recorded with *σ*^+^ helicity and *I*_m_ recorded with *σ*^−^ helicity were collected in a longitudinal geometry using photon energies tuned to the Mn L_3_-edge (642 eV), as shown in Fig. 5a. The symbols in Fig. 5b represent the experimental magnetic asymmetry defined by (*I*_p_-*I*_m_)/(*I*_p_ + *I*_m_) measured at the base temperature of 10 K. The experimental asymmetry data were fitted using the DYNA program^66,70,71^, and the best fit to the experimental data is shown by the solid line in Fig. 5b. All the parameters corresponding to the different slices of thin films used for fitting the soft X-ray resonant magnetic reflectivity data are listed in Table S1 in the supporting information. The asymmetry drops to zero at high angle and the fitting parameters (angles *ϕ* and *γ*) corresponding to the magnetization direction^70^ are 90°, indicating that the magnetic moments lie in the film plane. The reconstructed element-specific magnetization depth profile for Mn is shown in the inset of Fig. 5a. The magnetization depth profile clearly shows that the magnetization of the LSMO layer is not uniform close to the STO interface and slightly reduced but not reversed. The reason for the reduction in Mn moment in the bulk of the film is difficult to ascertain. Since we obtain near-bulk moments from the SQUID measurements, we attribute the reduced moments to the presence of magnetic domains not fully oriented in the direction of the original magnetic field. At the interface with the STO, the additional reduction is attributed to the presence of oxygen vacancies and the presence of a Mn^3+^-rich phase that suppresses the magnetization close to interface compared to the rest of the 30%-hole-doped LSMO layer. We normalized the magnetic moment in the magnetization depth profile by the remanent magnetization observed by SQUID VSM measurements, and the average calculated magnetic moment obtained from the resonance reflectivity fit is *m*_*tot*_ = 2.37 *µ*_*B*_/Mn, which is consistent with the XMCD result. In addition, we also simulated the experimental magnetic asymmetry for two different models consisting of a very thin (2 uc) LSMO dead layer (DL) at the interface in the first model and a 2 uc LSMO layer with (20%) negative magnetization (NM) at the interface in the second model. The experimental and simulated magnetic asymmetries and the extracted magnetic depth profiles for these two models are shown in Fig. 5c and d, e, respectively. The simulated magnetic asymmetry does not describe the experimental asymmetry in either case. The results from our element-specific XRMR measurements and the DYNA simulations clearly rule out the possibility of the presence of an inverse magnetized layer at the LSMO/STO interface.

## Conclusion

In summary, we have investigated the effects of interfacial nonstoichiometry on the magnetic properties of the epitaxial La_0.7_Sr_0.3_MnO_3_/SrTiO_3_(001) oxide heterostructure. We have demonstrated that the observation of the inverse hysteresis behavior in low coercivity LSMO thin films emanates from the remanent field of the superconducting SQUID-VSM solenoids and not from an antiferromagnetic intra-layer exchange coupling. A combined study of atomically resolved electron energy loss spectroscopy, element-specific X-ray magnetic circular dichroism and depth-sensitive polarized soft X-ray resonant magnetic reflectivity revealed the formation of a Mn^3+^enriched interfacial layer close to the SrTiO_3_ substrate, which is ferromagnetically coupled to the rest of the bulk-like top LSMO layer and leads to a proximity-induced ferromagnetism in Ti, which is mediated via interfacial oxygen vacancies. These results provide new insights to the understanding of the magnetism and spin structure of correlated oxide interfaces and also carry potential practical implications for spintronic and magnetic tunnel junction-based devices.

## Experimental Section

### Thin film growth and characterization

20 nm thick films of La_0.7_Sr_0.3_MnO_3_ were grown on single-crystalline [001]-oriented SrTiO_3_ substrates (with mixed SrO and TiO_2_ surface termination) by pulsed laser deposition (PLD) using a KrF excimer laser (248 nm). A single-phase stoichiometric La_0.7_Sr_0.3_MnO_3_ target was used for deposition. The films are grown at a substrate temperature of 750 °C under 350 mTorr of oxygen partial pressure and with 6 Hz repetition rate. During deposition, the energy density at the target surface was kept at ∼2.1 J/cm^2^. After deposition, the sample was cooled in an ambient oxygen environment (500 Torr). Structural characterization and reciprocal space mapping (RSM) were carried out using a high-resolution Bruker D8-Discover X-ray diffractometer equipped with a copper target. Macroscopic DC magnetization measurements were carried out in a 7-Tesla SQUID vibrating sample magnetometer (SVSM; Quantum Design Inc., USA) in the temperature range from 10 to 360 K. For correcting the remanent field of the superconducting solenoid of the SQUID-VSM, we used a high-purity paramagnetic Pd sample.

### STEM imaging and Electron Energy Loss Spectroscopy (EELS)

Cross-sectional TEM specimens (lamella) were prepared with a Xe^+^ plasma-based focused ion-beam system (TESCAN S8000X). The sample surface was coated with a protective layer of thin carbon (Sharpie marker) followed by Pt (deposited by an e-beam following the ion-beam) before the milling process was started to prevent damage during lamella preparation. In situ lift-off was used to mount the lamella on a TEM half-grid utilizing a Kleindiek probe manipulator. The FIB lamella was milled at 30 keV and thinned at 10 and 5 keV, and final cleaning was performed at 2 keV. HAADF (and ABF)-STEM and EELS measurements were carried out at 200 keV using a probe-corrected JEOL NEOARM. The convergence angle was kept at 25–29 mrad, and the condenser lens aperture was at 40 *µ*m with a camera length of 4 cm for STEM and 2 cm for EELS imaging, respectively. The GATAN GMS 3.5 software, a GATAN OneView IS camera and annular detectors were used to acquire and analyze the images along with a GATAN OneView IS camera and annular detectors. The stoichiometry and electronic structure at the interface were analyzed by atomically resolved electron energy loss spectroscopy (EELS).

### XMCD and soft X-ray Resonant Magnetic Reflectivity (XRMR)

To investigate the element-specific interfacial magnetism, temperature-dependent X-ray magnetic circular dichroism (XMCD) measurements at the Ti and Mn L-edges was carried out at the BESSY II undulator beamline UE46-PGM1. Absorption spectra using two different X-ray photon helicities (*σ*^+^ and *σ*^−^) were recorded in surface sensitive TEY mode (by measuring the sample drain current) and in bulk sensitive scattering mode, respectively, in a reflection geometry at 30° grazing incidence via a dedicated three-circle ultrahigh vacuum zero-field diffractometer^72^. X-ray resonant magnetic reflectivity (XRMR) spectra at the Mn and Ti L-edge resonance energies were recorded at the same beamline for the two X-ray photon helicities (*σ*^+^ and *σ*^−^) at a base temperature of 12 K. The measurements were carried out in the remanent state after cooling under a magnetic field from the paramagnetic state down to 12 K by placing a permanent magnet close to the sample during cooling. The XRMR data were simulated and fitted via the DYNA Matlab program^66,70,71^. To fit the data, the imaginary atomic scattering factors for Mn were derived from X-ray magnetic circular dichroism measurements at the Mn L-edge performed on the same LSMO thin film, whereas the real part was obtained from the Kramers-Kronig transformation.

## Supporting Information

In the supplementary information, we provide the details of angle-dependent magnetization hysteresis measurements performed on LSMO/STO(001) at 5 K. We present additional temperature-dependent magnetization hysteresis measurements on a second 20 nm LSMO thin film grown on STO(001). We present as well EELS spectra at the O K-edge across the LSMO/STO hetero-interface. We present the XMCD sum rule analysis to calculate the spin and orbital magnetic moments of the Mn ion. We show the TEY XAS measurement at the Ti L edge at the base temperature and the specular soft X-ray reflectivity at the Ti L-edge resonant energy at 10 K. We tabulate the fitting parameters of the soft X-ray resonant magnetic reflectivity (XRMR) data.

## Supplementary information

SUPPLEMENTARY INFORMATION

## Data Availability

The data that support the findings of this study are openly available at the PSI Public Data Repository database under http://doi.psi.ch/detail/10.16907%2F091627e3-7f93-47db-a302-b6196a6862cd.
