# Supplementary material for: Atomic scale determination of magnetism and stoichiometry at the La0.7Sr0.3MnO3/SrTiO3 interface: investigation of inverse hysteresis
Source: NPG Asia Mater. 2025 Mar 7;17(1):9. doi: 10.1038/s41427-025-00590-y (PMC11885156; doi:10.1038/s41427-025-00590-y)
Supplement: Supplementary file 1 — SUPPLEMENTARY INFORMATION [file 41427_2025_590_MOESM1_ESM.pdf]

Atomic scale determination of magnetism and stoichiometry at the $La_{0.7}Sr_{0.3}MnO_3/SrTiO_3$ interface: investigation of inverse hysteresis

Gyanendra Panchal,[1,2,a] Federico Stramaglia,[1] Pawan Kumar,[3,4] Enrico Schierle,[5] Klaus Habicht,[2,6] Carlos A. F. Vaz,[1] and Katharina Fritsch[2]

[1] *Swiss Light Source, Paul Scherrer Institut, Villigen, Switzerland*

[2] *Department Dynamics and Transport in Quantum Materials, Helmholtz-Zentrum Berlin für Materialien und Energie, Berlin, Germany*

[3] *Department of Materials Science and Engineering, University of Pennsylvania, Philadelphia, PA, USA*

[4] *Inter-university Microelectronics Center (IMEC), Leuven, Belgium*

[5] *Institute for Quantum Phenomena in Novel Materials, Helmholtz-Zentrum Berlin für Materialien und Energie, Berlin, Germany*

[6] *Institut für Physik und Astronomie, Universität Potsdam, Potsdam, Germany*

[a] Electronic mail: gyanendra.panchal@psi.ch

SUPPLEMENTARY INFORMATION

In this supplementary information, we provide the details of angle dependent magnetization hysteresis measurements performed LSMO/STO(001) at 5 K. We show additional temperature dependent magnetization hysteresis measurements on a second 20 nm LSMO thin film grown on STO(001). We show EELS spectra at the O K-edge across the LSMO/STO hetero-interface. We also show the XMCD sum rule analysis to calculate the spin and orbital magnetic moment of the Mn ion. We have shown the TEY XAS measurement at Ti L edge at base temperature and Specular soft x-ray reflectivity at the Ti L-edge resonant energy at 10 K. We have tabulated the fitting parameters of the soft x-ray resonant magnetic reflectivity (XRMR) data.

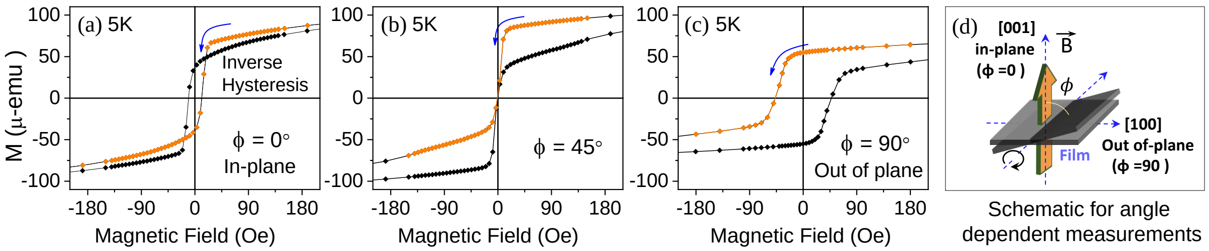

FIG. S1. Magnetization hysteresis at 5 K without remanence correction) as the function of angle ϕ (a) 0°, (b) 45°and (c) 90°. (d) Schematic for angle dependent magnetization measurements, ϕ represents the angle between sample plane and applied magnetic field direction.

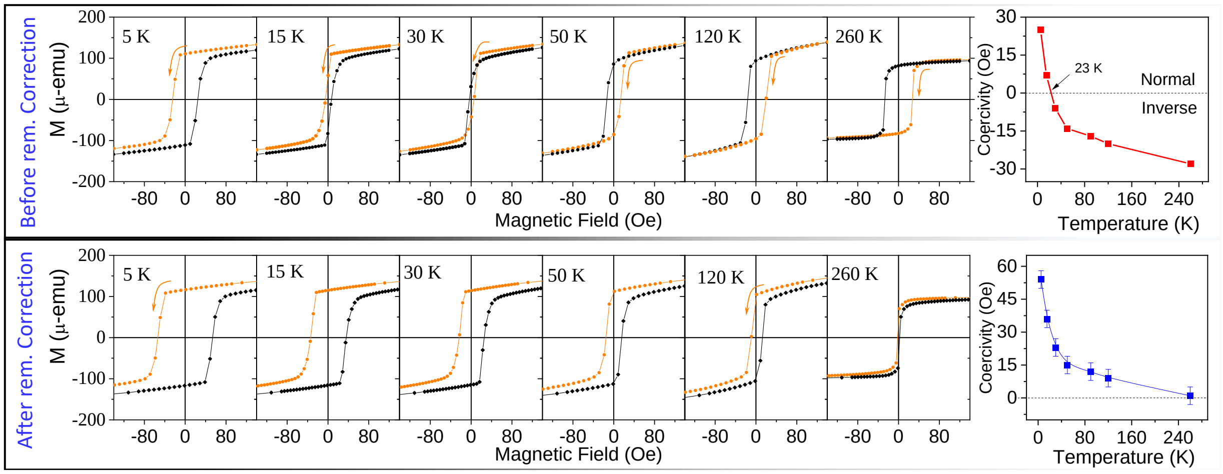

FIG. S2. Magnetization hysteresis of the second LSMO sample (grown under similar conditions) as a function of temperature in a temperature rage from 5 to 260 K. Top panel shows the transition from normal to inverse hysteresis around 25 K before correction of the remanence of the superconducting solenoid. Bottom panel shows the disappearance of inverse hysteresis after correction of the remanence of the superconducting solenoid.

We apply the XMCD sum rules[1,2] to obtain the spin and orbital moments for Mn:

$$\begin{aligned} m_{\mathrm{orb}} &= -\frac{4}{3}\frac{q}{r}(10-n_{\mathrm{3d}}) \\ m_{\mathrm{spin}} &= -\frac{6p-4q}{r}(10-n_{\mathrm{3d}})\left(1+\frac{7\,\langle T_z\rangle}{2\,\langle S_z\rangle}\right)^{-1} \end{aligned} \tag{1}$$

$$\begin{aligned} p &= \int_{L_3}(\mu_+-\mu_-)\mathrm{d}\omega \\ q &= \int_{L_3+L_2}(\mu_+-\mu_-)\mathrm{d}\omega \\ r &= \int_{L_3+L_2}(\mu_++\mu_-)\mathrm{d}\omega \end{aligned} \tag{2}$$

where $10-n_{\mathrm{3d}}$ is the number of holes in the 3d shell,[3] and r is the integrated area of the isotropic XAS spectra (summation of XAS spectra of two different helicities), p and q are the values of the integrated areas of the XMCD signal after the $L_{3,2}$ edge and between the L_3, L_2 edges, respectively:

We neglect the expectation value of magnetic dipole operator T_z and consider a spin correction factor $C = m_s/0.587$ to disentangle the overlap of the L_3 and L_2 parts in the spectrum.[4–6] The calculated value for the Mn spin magnetic moment is $m_s = 1.92\ \mu_B$/Mn

and for the orbital magnetic moment, $m_l = 0.01$ μ_B/Mn. After considering the projection of the magnetization along the x-ray beam, the calculated effective magnetic moment m_{eff} $= 2.22 \pm 20\%$ μ_B/Mn.

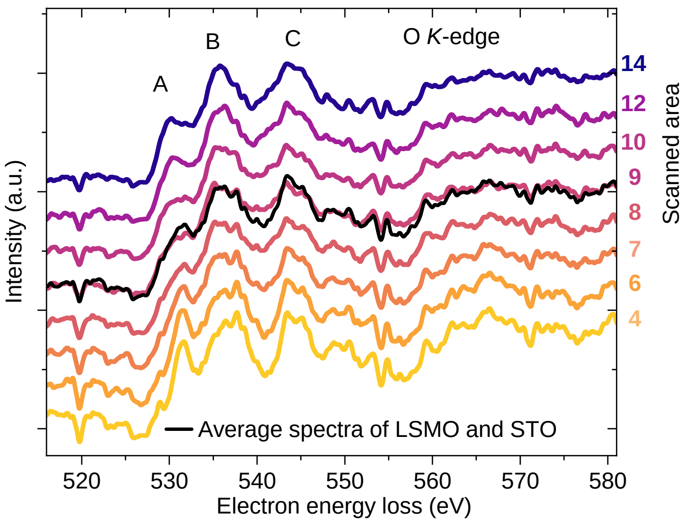

FIG. S3. (a) Layer-resolved EELS spectra at the O K-edge across the LSMO/STO hetero-interface; the number next to each spectrum represents the rectangular areas of the scanned regions used to collect the EELS spectra (see Fig. 3(a) of the main manuscript). Feature A is attributed to unoccupied O-2p states which are covalently intermixed with Mn-3d (in LSMO) or Ti-3d (in STO) orbitals. The broad features B and C are attributed to O-2p-derived states hybridized with La-5d/Sr-4f and Mn/Ti-4sp orbitals.[7,8] The black spectrum represents the average spectra of LSMO and STO, which is used to compare to the spectra of the interfacial layer and to illustrate the damping or suppression of the fine structure features A-C.[9]

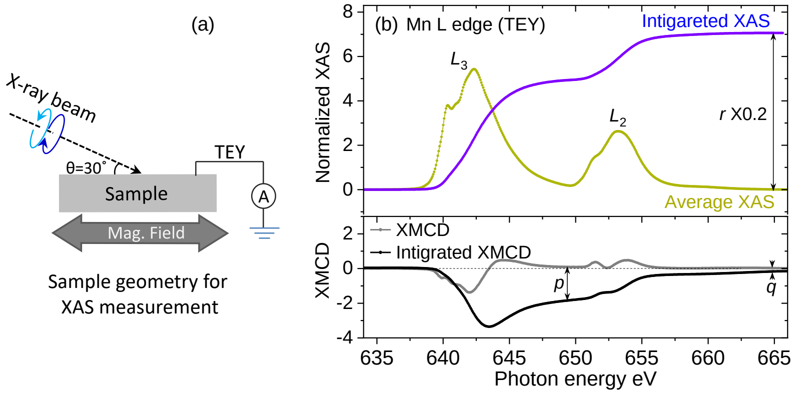

FIG. S4. (a) TEY XAS measurement geometry. (b) Normalized TEY XAS (after background subtraction) and XMCD spectra at the Mn L-edge together with the integrated XAS and XMCD signals.

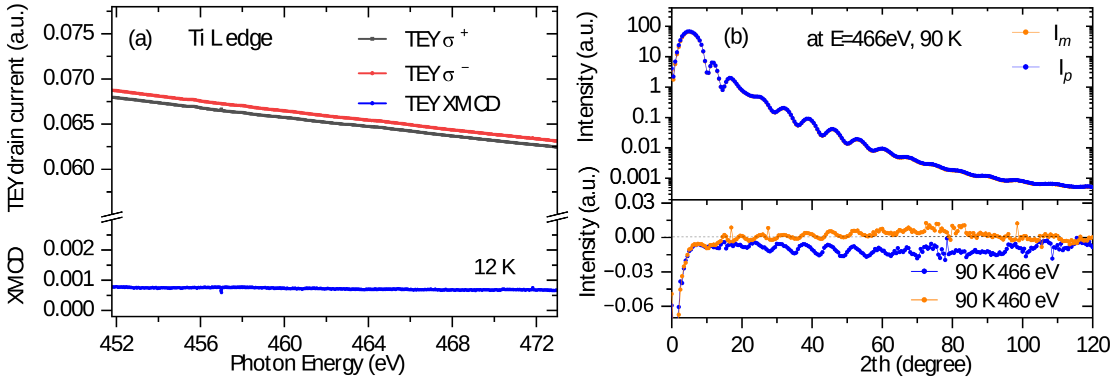

FIG. S5. (a) TEY XAS measurement at Ti L edge at the base temperature (12K). (b) Soft x-ray resonant reflectivity curves I_p and I_m at the Ti L_2-edge resonant energy 466 eV along with magnetic asymmetry at 466 and 460 eV at 90 K.

TABLE I. Fitting parameters of the soft x-ray resonant magnetic reflectivity (XRMR) data for the $La_{0.7}Sr_{0.3}MnO_3$thin film.

Layer	Thickness (Å)	Roughness (Å)	Magnetization (1=full moment at Mn ion)	Phi (°)	Gamma (°)
C	2.8	7.0	0.0	90	90
$La_{0.7}Sr_{0.3}MnO_3$	4.3	5.4	0.07	90	90
$La_{0.7}Sr_{0.3}MnO_3$	5.0	4.0	0.39	90	90
$La_{0.7}Sr_{0.3}MnO_3$	181.7	6.0	0.86	90	90
$La_{0.7}Sr_{0.3}MnO_3$	5.0	4.2	0.51	90	90
$La_{0.7}Sr_{0.3}MnO_3$	3.5	4.3	0.61	90	90
$SrTiO_3$-Sub.	1000	3.7	0.0	90	90

REFERENCES

[1]C. Chen, Y. Idzerda, H.-J. Lin, N. Smith, G. Meigs, E. Chaban, *et al.*, "Experimental confirmation of the x-ray magnetic circular dichroism sum rules for iron and cobalt," Physical review letters **75**, 152 (1995).

[2]J. Heidler, C. Piamonteze, R. Chopdekar, M. A. Uribe-Laverde, A. Alberca, M. Buzzi, *et al.*, "Manipulating magnetism in $La_{0.7}Sr_{0.3}MnO_3$ via piezostrain," Physical Review B **91**, 024406 (2015).

[3]T. Koide, H. Miyauchi, J. Okamoto, T. Shidara, T. Sekine, T. Saitoh, *et al.*, "Close correlation between the magnetic moments, lattice distortions, and hybridization in $LaMnO_3$ and $La_{1-x}Sr_xMnO_{3+\delta}$: doping-dependent magnetic circular x-ray dichroism study," Physical review letters **87**, 246404 (2001).

[4]C. Piamonteze, P. Miedema, and F. M. De Groot, "Accuracy of the spin sum rule in xmcd for the transition-metal l edges from manganese to copper," Physical Review B **80**, 184410 (2009).

[5]Y. Teramura, A. Tanaka, and T. Jo, "Effect of coulomb interaction on the x-ray magnetic circular dichroism spin sum rule in 3 d transition elements," Journal of the Physical Society of Japan **65**, 1053–1055 (1996).

[6]G. Shibata, K. Yoshimatsu, E. Sakai, V. R. Singh, V. K. Verma, K. Ishigami, *et al.*, "Thickness-dependent ferromagnetic metal to paramagnetic insulator transition in $La_{0.6}Sr_{0.4}MnO_3$ thin films studied by x-ray magnetic circular dichroism," Physical Review B **89**, 235123 (2014).

[7]P. Rajak, D. Knez, S. K. Chaluvadi, P. Orgiani, G. Rossi, L. Méchin, *et al.*, "Evidence of mn-ion structural displacements correlated with oxygen vacancies in $La_{0.7}Sr_{0.3}MnO_3$ interfacial dead layers," ACS Applied Materials & Interfaces **13**, 55666–55675 (2021).

[8]G. Panchal, D. M. Phase, V. R. Reddy, and R. J. Choudhary, "Strain-induced elastically controlled magnetic anisotropy switching in epitaxial $La_{0.7}Sr_{0.3}MnO_3$ thin films on $BaTiO_3$(001)," Phys. Rev. B **98**, 045417 (2018).

[9]D. A. Muller, N. Nakagawa, A. Ohtomo, J. L. Grazul, and H. Y. Hwang, "Atomic-scale imaging of nanoengineered oxygen vacancy profiles in $SrTiO_3$," Nature **430**, 657–661 (2004).
